# Identification of novel human receptor activator of nuclear factor-kB isoforms generated through alternative splicing: implications in breast cancer cell survival and migration

**DOI:** 10.1186/bcr3234

**Published:** 2012-07-23

**Authors:** Anastasios D Papanastasiou, Chaido Sirinian, Haralabos P Kalofonos

**Affiliations:** 1 Clinical and Molecular Oncology Laboratory, Division of Oncology, Department of Medicine, University of Patras, Rion 26504, Greece

## Abstract

**Introduction:**

The receptor activator of nuclear factor-kB (NF-kB) (RANK)/receptor activator of NF-kB ligand (RANKL) axis emerges as a key regulator of breast cancer initiation, progression and metastasis. RANK receptor is a tumor necrosis superfamily member, which upon ligand binding transduces a variety of survival, proliferation, differentiation and migration signals. The majority of these intracellular cues merge through the NF-kB transcription machinery.

**Methods:**

*TNFRSF11A *(RANK) variants were identified and cloned in mammalian expression vectors. Their expression was analyzed using real time PCR on RNA from normal tissue, cell lines and breast cancer specimens. Western blot analysis and immunofluoresence stainings were used to study expression and localization of protein isoforms in a panel of breast cancer cell lines and in transfected 293T cells. Luciferase assays were employed to assess the contribution of each isoform alone or in combinations on NF-kB activation. Isoform effect on cell survival after doxorubicin treatment was analyzed through MTT assay. Wound healing and transwell assays were employed to evaluate the effect of TNFRSF11A isoforms on migration of MDA-MB-231 and 293T cells.

**Results:**

We report the identification of three novel *TNFRSF11A *(RANK) variants, named *TNFRSF11A_Δ9*, *TNFRSF11A_Δ8,9 *and *TNFRSF11A_Δ7,8,9 *which result from the alternative splicing of exons 7 to 9. Interestingly, variant *TNFRSF11A_Δ7,8,9 *was found to be upregulated in breast cancer cells lines and its expression inversely correlated with tumor grade and proliferation index. *TNFRSF11A_Δ7,8,9 *encodes a 40-45 kDa protein, we named RANK-c, which lacks the transmembrane domain and most of the intracellular part of the wild type receptor. Furthermore, we showed that RANK-c could act as a dominant negative regulator of RANK-dependent NF-kB activation, affecting cell survival after apoptosis induction. In addition, RANK-c suppresses cell migration and represses the tumorigenic properties of invasive breast carcinoma cells.

**Conclusions:**

In this study, we provide evidence of a complex regulatory network of RANK receptor splice variants with a role in breast cancer. We identify that the RANK-c isoform is expressed in breast cancer samples and its expression reversely correlates with histological grade. Finally, isoform RANK-c seems to have the capacity to regulate signaling through wild type RANK and moreover to inhibit cell motility and migration of breast cancer cells.

## Introduction

Breast cancer is the most common malignancy, affecting one in eight women in North America and Europe [1,2]. Recently the receptor activator of NF-kB (RANK)/RANK ligand (RANKL) pathway was proven to be an important regulator of the mammary stem cell (MaSC) population [3,4] and mammary gland development [5-7], but also, a system with a key role in breast cancer initiation, progression [8,9] and metastasis [10,11].

The TNF receptor superfamily member, RANK (also called TNFRSF11A, ODFR, TRANCER, CD265), is a key regulator of T cell viability, dendritic cell function and survival [12,13], lymph node development [14] bone metabolism [15], and body temperature [16], through the interaction with its ligand, RANKL (also known as TNFSF11, ODF, TRANCE).

Despite the plethora of organs and cell types that depend on RANK function, little is known about the regulatory mechanisms that govern its functions both in normal cells and cancer cells. RANK expression is reported to be regulated at the transcriptional level through distinct extracellular cues, such as macrophage colony-stimulating factor (M-CSF) [17], 1alpha,25-dihydroxyvitamin D3 [18], follicle stimulating hormone (FSH) [19], lipopolysaccharide (LPS) [20] and also at the post-transcriptional level through the action of IL-3 [21]. In addition, a recent report provides evidence of RANK receptor shedding from the cell surface in the mouse [22].

RANK stimulation leads to activation of the nuclear transcription complex NF-kB in RANK-expressing human T cells and transfected 293T cells [12], through its long (383 amino acids) cytoplasmic domain. The NF-kB activation is dependent on the interaction of TNF receptor-associated factor (TRAF) adaptor proteins with specific modules and residues of the intracellular part of the RANK receptor, and partial or complete deletion of these segments alter RANK signaling and thus NF-kB activation [23]. NF-kB plays a central role in several physiological and pathophysiological processes. It participates in the regulation of cell cycle progression through its effects on cyclin D1 expression [24] and most importantly it has been implicated in the regulation of cell death through its ability to regulate the expression of cellular factors that affect the apoptotic threshold [25].

Alternative splicing (AS) is a major post-transcriptional modification that occurs in 92 to 94% of human pre-mRNA transcripts, through which individual mammalian genes often produce multiple mRNA and protein isoforms that may have related, distinct or even opposing functions [26]. More specifically, many cytokine receptors such as IL6R, fibroblast growth factor receptor (FGFR), IL15Ra, IL1RII, erythropoietin receptor (EPOR), gp130, IL17R, IFNAR1 and most importantly CD40, another TNF receptor (TNFR) family member with high similarity to RANK, regulate part of their functions through isoforms produced by AS [27,28].

In this study, we identified three novel variants of *TNFRSF11A*, named *TNFRSF11A_Δ9*, *TNFRSF11A_Δ8,9 *and *TNFRSF11A_Δ7,8,9 *which result from the alternative splicing of exons 7 to 9. Interestingly, variant *TNFRSF11A_Δ7,8,9 *was highly upregulated in breast cancer samples and seems to encode a 40 to 50 kDa protein, which we named RANK-c. By characterizing the molecular and cellular properties of RANK-c in conjunction with the other isoforms and the wild type receptor, we showed that this novel isoform acts as a dominant negative regulator of NF-kB through wild type RANK, with consequences for cell survival and apoptosis. In addition, RANK-c seems to be a suppressor of cell migration and represses the tumorigenic properties of invasive breast carcinoma cells.

## Materials and methods

### Cell lines, antibodies and reagents

All cell lines were purchased from the American Type Culture Collection (ATCC). MDA-MB-468, SKBR3, U87, M059K, HeLa, Caco2, HT-29, 293T cells were grown in DMEM with 10% fetal bovine serum (FBS). MDA-MB-231, MCF-7 cells were cultured in Eagle's minimum essential medium (EMEM) with 10% fetal bovine serum (FBS). T47D, HT-29, A549, THP-1 and Jurkat cells were grown in Roswell Park Memorial Institute medium (RPMI) with 10% FBS. MCF10A cells were cultured in DMEM F12 with 5% FHS. Human skin fibroblast cell line (1BR3) was purchased from European Collection of Cell Cultures (ECACC) and cultured in EMEM with 15% FBS. Peripheral blood mononuclear cells (PBMCs) were isolated from whole blood of three healthy donors by centrifugation on Ficoll-Paque (Amersham Biosciences, Uppsala, Sweden). The following primary antibodies were used: anti-human RANK antibodies: (AF683, 1 μg/ml, extracellular domain R&D systems, Abingdon, UK), (sc-9072, 0.2 μg/ml, intracellular domain aa 317-616, Santa Cruz Biotechnology Inc., Santa Cruz, CA, USA), anti-actin (C4, MAB1501R, 0.01 μg/ml, Chemicon, EMD Millipore, Billerica, MA, USA) and mouse monoclonal anti-HA (sc-57592, Santa Cruz Biotechnology Inc.). Secondary antibodies were Alexa Fluor^® ^568 donkey anti-goat (A-11057, Molecular Probes Inc., Eugene, OR, USA) Alexa Fluor^® ^568 goat anti-mouse (A-11004, Molecular Probes), goat anti-mouse IgG FITC (F0257, Sigma, St. Louis, MO, USA), goat anti-rabbit IgG HRP (12-348, Millipore, Temecula, CA, USA) and goat anti-mouse IgG HRP (12-349, Millipore). Recombinant human sRANKL was used in a final concentration of a 0.1 -1 μg/ml (Acris Antibodies GmbH, Herford, Germany).

### Tissues samples and histological examination

Breast carcinoma FFPE (Formalin Fixed Paraffin Embedded) samples were retrieved from the archives of the Department of Pathology, General Hospital of Patras, Agios Andreas, Greece. The selected cases comprised invasive ductal breast carcinoma of grade 1 (three patients), grade 2 (ten patients) and grade 3 (eight patients). Histopathological grading (Nottingham grading system) and immunohistochemistry evaluation of protein markers were done as part of the routine diagnostic procedure. No ethical approval and patient inform consent was required for the present study, according to the scientific and bioethics committee of the General Hospital of Patras, Agios Andreas.

### RNA isolation, cDNA synthesis, PCR and qRT-PCR

Total RNA from normal brain, bone marrow, thymus, PBMCs, breast, cell lines and samples from paraffin-embedded tissues was obtained from Biochain (Hayward, CA, USA) or isolated using Absolutely RNA^® ^Purification kit (Stratagene, La Jolla, CA, USA). cDNA synthesis was carried out using the Superscript III cDNA synthesis kit (Invitrogen, Carlsbad, CA, USA) from 1μg of total RNA. PCR was performed using the FastStart High Fidelity PCR System (Roche, Mannheim, Germany). RANK variant mRNA relative expression levels were assessed, using gene-specific primers (Additional file 1) and the One-Step quantitative real time (qRT)-PCR kit KAPPA SYBR FAST (Kappabiosystems, Mowbray, South Africa) with the Rotor-Gene 3000 (Corbett Research, Sydney, Australia). Relative expression level of the gene of interest was calculated with the comparative 2*^ΔΔCt ^*method, where ΔC_t _= target C_t _- control C_t_, ΔΔC_t _=ΔC_t _target - ΔC_t _calibrator.and all samples were normalized to the glyceraldehyde 3-phosphate dehydrogenase (GAPDH) gene for PCR and to GAPDH and human aminolevulinate delta-synthase 1 (ALAS1) (QuantiTect Primer Assay, Qiagen GmbH, Hilden, Germany) genes for qRT-PCR. All experiments were independently performed in duplicate three times, each time using 1μg of template RNA. All experimental procedures that involved archived paraffin-embedded human tissue specimens did not need any patient consent and were conducted according to the principles laid down by the Declaration of Helsinki.

### Plasmids and transfection

PBMC cDNA was used to amplify full-length RANK variants using primers P4 and P5 (Additional file 1). The PCR products of the expected size were ligated into the pGEM^® ^-T Vector Systems (Promega Corp., Madison, WI, USA) and sequenced (VBC, Austria). Inserts from every pGEMT-RANK variant was digested with ApaI-NotI restriction enzymes and re-ligated into pCDNA3.1/Hygro(-) (Invitrogen).

The primers P6 and P7 (Additional file 1, Table S1), containing restriction sites (HindIII and BamHI) were used to amplify the RANK-c (*TNFRSF11A_Δ7,8,9*) open reading frame (ORF). The PCR product was digested and ligated into pEGFP (plasmid enhanced green fluorescent protein) vector (Clontech, Mountain View, CA, USA) to produce RANK-c fused to green fluorescent protein (GFP). Human influenza hemagglutin epitope (HA)-tagged wild type (wt) RANK and RANK-b was generated by introducing the pCDNA3.1-RANK isoform plasmids, one repeat of the HA at amino acid position 33 of the wt RANK.

All PCR products were fully sequenced (VBC, Austria). Cell transfections were performed using TurboFect™ *in vitro *Transfection Reagent (Fermentas GmbH, Germany) according to the manufacturer's instructions.

### Western blotting

After 48h of transfection 293T cells were harvested and lysed directly in SDS-PAGE loading buffer and boiled. The supernatants from each well were collected after an additional 24 h treatment with DMEM/1% FBS and concentrated 4-fold in a Vivaspin 500 μl centrifugal filter unit or left unconcentrated. Cell lysates and cell culture supernatants were loaded onto a 10% acrylamide gel, transferred onto polyvinylidene difluoride (PVDF) membrane (Millipore). Total Protein Western Blot (W1235086)from a panel of human breast cancer tissues collected from three different donors, benign lesions and normal tissue, was purchased from Biochain.

### Immunofluorescence

The 239T cells growing on polylysine-covered coverslips were transiently transfected. After 48 h, the cells were fixed in 4% paraformaldehyde for 10 minutes and processed as previously described [29]. HA-tagged molecules were visualized with the use of anti-HA (0.01 μg/ml) and Alexa Fluor^® ^568 (0.005 μg/ml). Images were recorded on a Nikon Eclipse TE 2000-U inverted microscope using 60×/1.40 oil and 40×/0.75 lenses. ImageJ (Bethesda, Maryland, USA) software was used to process the images.

### NF-kB reporter assay

The 293T cells were seeded at a density of 1×10^4 ^cells/well in 24-well plates, and transiently transfected with a total of 140 ng plasmid DNA (empty vector was used where appropriate, to keep the total amount of DNA used constant). The NF-kB reporter construct pNF-κB-luc (Agilent Technologies, Santa Clara, CA, USA) was used at a concentration of 10 ng/well. To normalize and correct for transfection efficiency, 7ng/well of pRL-TK vector (Promega) was co-transfected. At 16h post-transfection, RANKL was added to the cells for another 24h. Luciferase assays were performed with the Dual-Luciferase Reporter assay system (Promega). Relative NF-kB/luciferase activities were normalized to Renilla luciferase expression levels and are reported as mean values (± SD) from duplicate transfections.

### Cell proliferation assay

To determine whether RANK-c affect the proliferation of MDA-MB-231 and 239T cell lines, the 3-(4,5-dimethylthiazol-2-yl)-2,5-dimethyltetrazolium bromide(MTT) assay was used. Briefly, cells were plated at a density of 2 × 10 ^4^cells per well in 24-well tissue culture plates and transiently transfected with the appropriate plasmids. At 16 h post-transfection the medium was replaced and recombinant RANKL and/or doxorubicin were added. Cell proliferation was measured 24 h and 48 h after addition of RANKL and/or doxorubicin using the MTT (3-(4,5-dimethylthiazol-2-yl)-2,5-dimethyltetrazolium bromide) assay, as previously described [30].

### Flow cytometry

The 293T transfected cells (up to 1x10^6^) with a total of 1μg plasmid DNA (0.5 μg wt Rank + 0.5 μg mock vector, 0.5 μg Rank-c + 0.5 μg mock vector, 0.5 μg wt Rank + 0.5 μg Rank-c and 0.5μg wt Rank + 250 ng Rank-c + 250 ng mock vector) were resuspended in 100μl 1xPBS/2%FBS/2mM EDTA and left for 10 minutes at RT (Room Temperature) The cells were then incubated with the mouse monoclonal anti-HA (0.004 μg/μl) for 30 minutes at RT. After three washes with PBS/FBS/EDTA, the cells were incubated with goat anti-mouse Ig fluorescein isothiocyanate (FITC) for 10 minutes. The cells were then washed twice with PBS and resuspended in 300 μl of ice cold PBS. Flow cytometry was performed on an EPICS-XL (Beckman Coulter, Inc.). Data was analyzed with FlowJo 7.6.5 software (Tree Star, Inc. Ashland, OR, USA).

### Scratch motility (wound-healing) assay

Cells were plated in a six-well plate at a concentration of 5 × 10^5 ^per well and transiently transfected. At 16h post-transfection the medium was replaced with 1% FBS and cells were left to grow to 90% confluence. The monolayer was scratched with a yellow pipette tip and photographed (time 0). After 24 h, plates were photographed at the marked spots.

### Migration assay

The migration assay was performed using Transwell chambers (Corning Inc., NY, USA) with 8-μm pore membranes. MDA-MB-231 cells were transiently transfected for 16 h and then left in full medium for 24 h. Cells were trypsinized, resuspended and plated (2 × 10^5^) into the upper chamber containing serum-free medium, and allowed to migrate toward 700 μl EMEM supplemented either with 1% FBS alone or recombinant RANKL (1 μg/ml). After 6 h, the upper chamber was scraped using a cotton swab and the cells on the lower surface of the membrane were fixed with 4% paraformaldehyde and stained with Giemsa. Experiments were done in triplicate and the data are presented as mean values (± SD). Three randomly chosen fields of stained cells were counted and averaged.

### Statistical analysis

Differences between groups and controls were tested by the Student's *t*-test or one-way analysis of variance (ANOVA). To evaluate weather RANK-c mRNA levels correlate with tumor histological grade we used the Mann-Whitney-Wilcoxon test. Possible correlations of protein markers (p53, Ki-67, ER, PR, Her2) and RANK-c mRNA levels were tested using Spearman's *r *correlation coefficient. All data were analyzed with the SPSS program (SPSS^® ^release 15.0, Chicago, IL, USA). Any *P-*value less than 0.05 was considered statistically significant.

## Results

### Identification of novel *TNFRSF11A *splice variants differentially expressed in normal tissue and cancer cell lines

**Figure 1:**
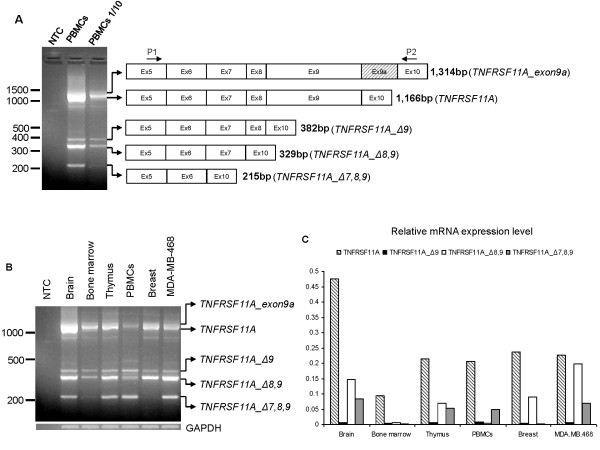
**Identification and mRNA expression profile of *TNFRSF11A *gene variants in normal tissue**. **A**. Agarose gel electrophoresis of the PCR products using primers P1 and P2 on cDNA from human peripheral blood mononuclear cells (PBMCs) and the graphical representation of the splice products identified. **B**. Agarose gel electrophoresis of PCR products depicting *TNFRSF11A *variant distribution from a panel of human normal tissue RNAs and MDA-MB-468 as a control. **C**. Quantitative RT-PCR of the novel splice variants and wild type (wt) receptor activator of NF-kB (RANK) from a panel of human normal tissue RNAs. Data normalization was carried out against the *GAPDH *housekeeping gene.

To examine whether RANK receptor has isoforms that are generated by alternative splicing, we isolated total RNA from untreated PBMCs and used it for cDNA construction. The amplification of the intracellular part of the RANK coding sequence by PCR using primers flanking exons 6 to 9 (P1 on exon 5 and P2 on exon 10) (Additional file 1) revealed the constitutive expression of five transcripts (five bands on the agarose gel) by non-activated PBMCs, with approximate sizes of 1,300, 1,100, 400, 350 and 210 bp (Figure 1A). Subsequent cloning and sequencing of these fragments identified the approximately 1,300 bp (1,314 bp) band as the wt *TNFRSF11A *transcript with the addition of a novel exon of 148 bp named exon 9a [HE659518: EMBL] between the already known exons 9 and 10 (Additional file 2). The approximately 1,100 bp (1,166 bp) fragment was identified as the wt *TNFRSF11A *(wt RANK), whereas the three smaller fragments were truncated versions of the *TNFRSF11A *gene. The approximately 400 bp (382 bp) fragment (*TNFRSF11A_Δ9*) lacks exon 9 (784 bp deletion); the approximately 350 bp (329 bp) fragment (*TNFRSF11A_Δ8,9*) has a deletion of exons 8 and 9 (837 bp deletion) and the smallest fragment (*TNFRSF11A_Δ7,8,9*) misses exons 7, 8 and 9 (951 bp deletion) (Figure 1A).

To determine the distribution of the *TNFRSF11A *transcripts in adult human tissues, we performed semi-quantitative RT-PCR using primers P1 and P2 (Figure 1B) and qRT-PCR (Figure 1C) employing a set of primer pairs designed specifically for every splice variant (Additional file 1). Most of the splice isoforms were detected in brain, bone marrow, thymus, PBMCs and breast, whilst the *TNFRSF11A_Δ7,8,9 *variant was absent from bone marrow and breast. The *TNFRSF11A_Δ9 *transcript was expressed at low levels in all tissue specimens tested, whereas *TNFRSF11A_Δ8,9 *transcript was abundantly expressed only in brain, thymus and breast. The wt RANK was always expressed in all samples tested.

**Figure 2:**
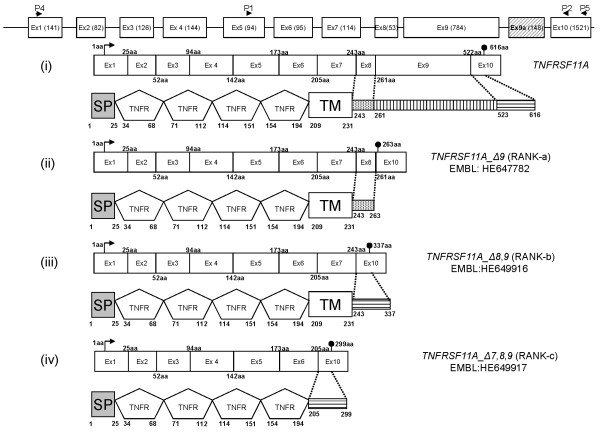
**Tumor necrosis factor receptor superfamily, member 11a *(TNFRSF11A) *gene organization, splice pattern, protein domain architecture and the positions of the primers used (arrowheads)**. On the top: the structure of the *TNFRSF11A *gene with the addition of the new exon 9a. Inside numbering represents exon length (nucleotides). Figure shows the exon organization of each alternative spliced isoform with a representative protein structure: (i) the wt TNFRSF11A (receptor activator of NF-kB, RANK), numbering in exon boundaries indicates amino acid residue at the splice junction, (ii) the 263-aa-residue-long RANK-a (TNFRSF11A_Δ9), encompassing SP, four extracellular TNFR repeats, TM, and 20 aa of intracellular tail (from exon 8), (iii) the 337-aa-residue-long RANK-b (TNFRSF11A_Δ8,9) encompassing SP (signal peptide), four extracellular tumor necrosis factor receptor (TNFR) repeats, transmembrane domain (TM) and 94 aa intracellular tail (from exon 10) and (iv) the 299-aa-residue-long RANK-c (TNFRSF11A_Δ7,8,9) encompassing SP, four extracellular TNFR repeats and 94 aa from exon 10. The arrows indicate the translation start site and the black circle represents a stop codon. The putative protein products of exons 8, 9 and 10 are depicted as a doted, vertically and horizontally lined box, respectively.

We sought to clone the full-length mRNAs of *TNFRSF11A *(wt RANK), *TNFRSF11A_Δ9, TNFRSF11A_Δ8,9 *and *TNFRSF11A_Δ7,8,9*. To that end we used primers P4 and P5, (Figure 2 andAdditional file 1) flanking the initiation start codon (ATG) in exon 1 and the termination codon (TGA) in exon 10 and cloned the bands from the anticipated molecular weights in TA vectors. After sequencing of the cloned fragments, we identified one clone encoding for the full-length wt *TNFRSF11A *and three full-length clones encoding *TNFRSF11A *variants (HE647782, HE649916, HE649917: EMBL) (Figure 2) (variant *TNFRSF11A_exon9a *could not be identified in full-length). The wt *TNFRSF11A *and the three full-length splice variants were subcloned into mammalian expression vectors and transiently transfected into 293T cells. Western blot analysis of the cell pellets and cell culture supernatants was performed, as well as immunofluorescence (IF) stainings for isoform localization (Additional file 3).

Thus, three of the novel variants were cloned as full-length molecules and almost all *TNFRSF11A *novel variants are expressed along with wt *TNFRSF11A *in all tissues tested. Moreover, their ratio depended on tissue type, suggesting a tissue-dependent effect of *TNFRSF11A *variants, and especially *TNFRSF11A_Δ7,8,9*, on *TNFRSF11A *properties. In addition, the absence of *TNFRSF11A_Δ7,8,9 *variant from normal breast in conjunction with the observed expression of this transcript in MDA-MB-468 human breast cancer cell line prompted us to further focus on the possible roles of the *TNFRSF11A *variants in breast cancer.

### *TNFRSF11A_Δ7,8,9 *variant is expressed in breast cancer cell lines and breast tumors

Because of the difference in expression observed between normal breast and breast cancer cells (MDA-MB-468) for *TNFRSF11A_Δ7,8,9*, we further investigated its expression profile. Total RNA from MCF10A, T47D, MDA-MB-231, SKBR3, MCF-7, MDA-MB-468 cells and a panel of cell lines (Additional file 4) was used to determine mRNA expression by both RT-PCR and qRT-PCR.

**Figure 3:**
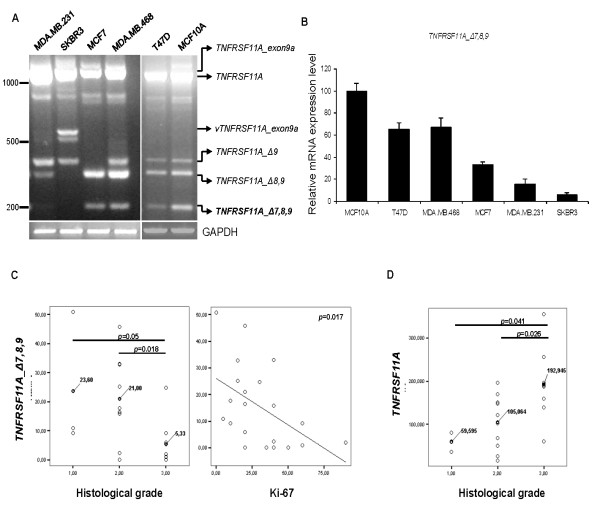
**Expression of mRNA encoding tumor necrosis factor receptor superfamily, member 11a *(TNFRSF11A*, wild type receptor activator of NF-kB, wt RANK), *TNFRSF11A_Δ9 *(RANK-a), *TNFRSF11A_Δ8,9 *(RANK-b) and *TNFRSF11A_Δ7,8,9 *(RANK-c) in breast cancer cell lines and breast cancer samples**. **A**. Agarose gel electrophoresis (2%) of PCR products using primers P1 and P2 depicting *TNFRSF11A *and the identified alternative splice variants in MDA-MB-231, SKBR3, MCF7, MDA-MB-468, T47D and MCF10A breast cancer cell lines. *vTNFRSF11A_exon9a *was observed only in SKBR3 and after cloning and sequencing was identified as a *TNFRSF11A *variant encompassing exons 6, 7, 8, 9a and 10. Splice variant *TNFRSF11A_Δ7,8,9 *is in bold. **B**. *TNFRSF11A_Δ7,8,9 *(RANK-c) relative mRNA expression levels in a panel of breast cancer cell lines and the non-tumorigenic epithelial cell line MCF10A; values obtained for MCF10A were set to 100. **C**. Relative mRNA expression levels of variant *TNFRSF11A_Δ7,8,9 *(RANK-c) in a panel of formaldehyde-fixed paraffin embedded (FFPE) invasive ductal breast carcinoma samples analyzed against tumor histological grade and proliferation index Ki-67, respectively. **D**. Relative mRNA expression levels of *TNFRSF11A *(wt RANK) in the same panel of invasive ductal breast carcinoma samples plotted against tumor histological grade.

While wt *TNFRSF11A *expression was detected in all breast cancer cell lines tested, the *TNFRSF11A_Δ7,8,9 *variant was observed only in MCF10A, T47D, MCF-7 and MDA-MB-468 cell lines when conventional PCR and gel electrophoresis were employed (Figure 3A). In the same way, the use of qRT-PCR revealed the down-regulation of the *TNFRSF11A_Δ7,8,9 *transcript 1.5- to 16.0-fold relative to the non-tumorigenic epithelial cell line MCF10A, in the breast cancer cell lines T47D, MCF-7, MDA-MB-468 and especially in the more aggressive MDA-MB-231 and SKBR3 (Figure 3B).

To assess the mRNA expression of the *TNFRSF11A_Δ7,8,9 *variant in breast cancer tissues and correlate its levels with protein markers, total RNA from 21 FFPE samples of invasive ductal breast carcinoma tumors was directly used for qRT-PCR with transcript-specific primers, as above. We observed that mRNA expression levels of the *TNFRSF11A_Δ7,8,9 *inversely correlated with tumor histological grade in all tumor samples tested (*P *= 0.011). In addition, further statistical analysis showed that the expression levels of *TNFRSF11A_Δ7,8,9 *variant decreased significantly between groups of grade 1 and 3 (*P *= 0.05) and grade 2 and 3 (*P *= 0.018) (Figure 3C). In contrast, *TNFRSF11A *mRNA expression levels showed a tendency to increase as the histological grade increased (Figure 3D). Finally, among protein markers tested, proliferation index Ki-67 showed an inverse correlation with *TNFRSF11A_Δ7,8,9 *expression (*P *= 0.017) indicating that as breast cancer evolves to a more aggressive disease state the expression of the *TNFRSF11A_Δ7,8,9 *diminishes (Figure 3C and Additional file 4).

### *TNFRSF11A_Δ7,8,9 *variant encodes RANK-c, a novel RANK protein isoform, observed in cell lines and tumor samples

The novel *TNFRSF11A_Δ7,8,9 *variant codes for a 299-amino-acid (aa) RANK protein, which lacks amino acids 206 to 522 of the wt RANK. Specifically, the novel isoform lacks the transmembrane domain (212-233aa) and a large portion of its intracellular part (234-522aa) that includes important functional sites (for example, 353-PSQPT-357) of exon 9 [31]. The lack of a transmembrane segment indicates that probably this isoform is excluded from the membrane and it is either in the cytoplasm or it is secreted. The product of *TNFRSF11A_Δ7,8,9 *variant in this study was named RANK-c, because it lacks three exons and was identified as the third and smaller migrating band in our initial cloning experiments. In the same way the putative protein products of *TNFRSF11A_Δ9 *and *TNFRSF11A_Δ8,9 *were named RANK-a and RANK-b, respectively.

Western blot analysis of native MCF-7 and MDA-MB-231 cells and *TNFRSF11A_Δ7,8,9 *cDNA-transfected 293T cells, using the AF683 (R&D systems) anti-RANK antibody directed against the extracellular domain of RANK, revealed the presence of a 40-45 kDa endogenous protein (Figure S2D in Additional file 3), which migrates exactly the same way as exogenous RANK-c does. Both transfected and native forms of RANK-c seem to migrate as a double band at a higher molecular mass than that theoretically predicted (29 kDa) possibly due to the presence of cysteine-rich regions as well as post-translational modifications, such as glycosylation.

In addition, a commercial ready-to-use western blot (Biochain) was employed for identification of RANK-c in protein lysates from a panel of representative breast tumors, benign lesions and normal tissue. Hybridization with anti-RANK antibody (AF683, R&D systems) revealed a band migrating approximately at 45 kDa, resembling RANK-c (Figure S2F in Additional file 3). Importantly, the protein expression profile of the commercial blot resembled that of the mRNA, where RANK-c is absent from normal breast but is upregulated in low-grade and declines in high-grade tumors. Nonetheless, a definite identification of a native RANK-c protein needs further experimental confirmation and probably development of more sensitive analytical tools.

### RANK-c protein isoform is a dominant negative regulator of RANK-induced NF-kB activation

From the mRNA expression analysis in various cell lines it appeared that *TNFRSF11A *variants could co-exist in the same cell population, raising the possibility of a role in the fine-tuning of RANK-dependent downstream signaling. A major downstream ultimate target of RANK signaling is the NF-kB transcription factor which, upon activation, translocates to the nucleus and promotes the transcription of numerous target genes, conferring survival advantages and anti-apoptotic capacity in cells [25].

In order to characterize the role of RANK isoforms and especially RANK-c (*TNFRSF11A_Δ7,8,9*) in the activation of NF-kB, we established a dual Luciferase/NF-kB-responsive reporter system. The 293T cells were transiently transfected with the indicated RANK isoform or were mock transfected in combination with the Luc-NF-kB-responsive plasmid and incubated with recombinant RANK ligand or left untreated.

**Figure 4:**
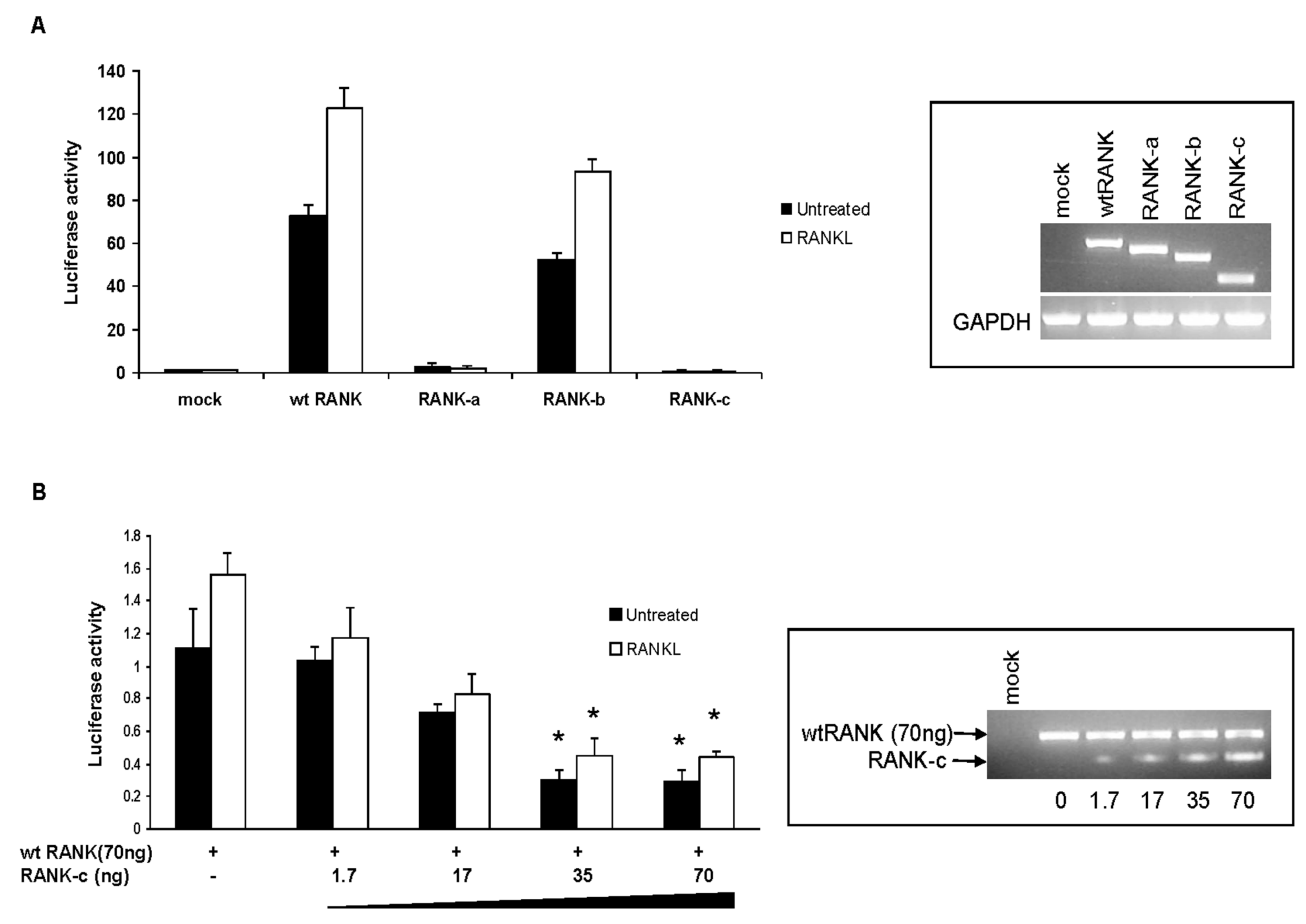
**Receptor activator of NF-kB (RANK)-c tumor necrosis factor receptor superfamily (*TNFRSF-11A_Δ7,8,9*) isoform has a dominant negative effect on RANK/RANK ligand (RANKL)-dependent NF-kB activation**. **A**. Expression vectors containing wild type (wt) RANK or its isoforms were transfected in combination with a NF-kB-responsive luciferase reporter construct into 293T cells. Luciferase activity in mock-transfected cells was set to 1. RNA was extracted from transfected cells and mRNA expression levels of the indicated isoforms were quantified by PCR (right panel) **B**. Expression vectors containing RANK and RANK-c were co-transfected (at the indicated amounts) with a NF-kB-responsive luciferase reporter construct into 293T cells. *P *< 0.001 between asterisk-indicated samples and wt RANK luciferase levels (untreated and RANKL-treated, respectively). RNA was again extracted from transfected cells and mRNA expression levels of the indicated isoforms were quantified by PCR (right panel).

As expected, wt RANK could induce the highest luciferase activity, and was highly active even in the absence of RANKL, followed by the exon 8, 9 truncated form of RANK (RANK-b), which also showed elevated luciferase activity in the absence of stimulus. The RANK-a (*TNFRSF11A_Δ9*) isoform, which resembles a decoy RANK receptor lacking most of the intracellular domain, did not upregulate luciferase activity either alone or with the addition of RANK ligand. The same result, on luciferase activity, was observed with the RANK-c isoform (Figure 4A). In accordance with previous results [23,32], these data indicate that the RANK receptor is a very potent inducer of NF-kB and when overexpressed in cells there is no requirement for RANKL stimulation, for downstream signaling to occur.

Next we aimed to characterize the effect of the co-expression of RANK isoforms on NF-kB activation, because of the observed co-existence of RANK transcripts in the same cell population. Surprisingly, when co-transfecting wt RANK with the RANK-c isoform (*TNFRSF11A_Δ7,8,9*), there was a significant (*P *< 0.001) downregulation of luciferase activity compared to wt RANK (Figure 4B). In contrast, none of the other isoforms were capable of inhibiting NF-kB activation through wt RANK, as RANK-c did (Figure S4A in Additional file 5). Furthermore, isoform RANK-b (*TNFRSF11A_Δ8,9*), which is the putative membrane-bound form of RANK-c, was unable to inhibit wt RANK-induced NF-kB activation (Figure S4B in Additional file 5). At the same time RANK-c could not inhibit the RANK-b induced NF-kB activation, to the same extent as for wt RANK, indicating an isoform-specific effect (Figure S4C in Additional file 5). The same inhibitory phenomenon was also observed when a GFP-RANK-c construct was used in combination with wt RANK receptor (Figure S4D in Additional file 5).

Thus, according to the luciferase data RANK-c is a putative dominant negative regulator of RANK signaling through NF-kB transcription factor machinery, showing a specific effect on the wild type receptor.

### Reduction of the wild type RANK on the cell surface by expression of RANK-c isoform

**Figure 5:**
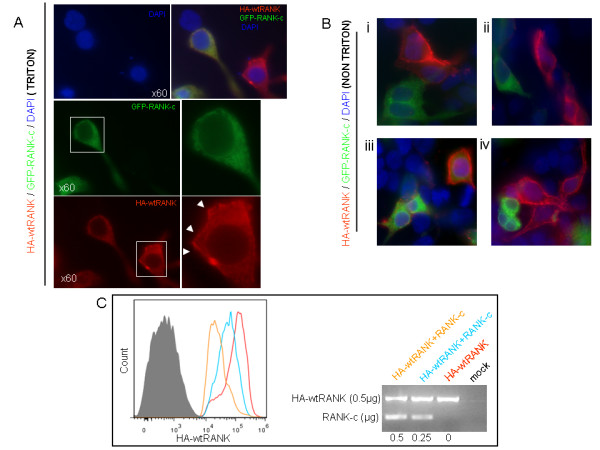
**Receptor activator of NF-kB (RANK)-c reduces the levels of wild type (wt) RANK on the cell surface**. **A**. 293T cells transiently transfected with wt HA-RANK in combination with green fluorescent protein (GFP)-RANK-c. Cells were fixed and stained with the use of triton 0.01% for permeabilization of the cell membrane. Representative recording of a merged field (upper panel) showing the membrane localization of a single-positive human influenza hemagglutin epitope (HA)-RANK (red) cell and the cytoplasmic retention of HA-RANK when co-expressed with GFP-RANK-c. In the lower panel the split field of the same field in more detail. White boxes indicate the area of focus. The arrowheads depict the clear membrane localization of wt RANK when expressed alone in a cell. **B**. Four representative fields of unpermeabilized (non triton) cells fixed and stained as above. In the capture only merged fields are depicted. Split channels can be found in Additional file 6. HA molecules were visualized with the use of anti-HA from Santa Cruz (sc-57592). **C**. 293T cells were transfected with HA-wt RANK alone or in combinations with RANK-c and analysed by flow cytometry. Isotype control from mock-transfected cells (grey-filled) wt RANK is depicted by the red line and co-transfections of wt RANK and increasing amounts of RANK-c (0.25 and 0.5μg) are depicted by blue and yellow lines, respectively. RNA was extracted from transfected cells after flow cytometry and mRNA expression levels of the indicated isoforms were quantified by PCR (right panel).

To investigate the mechanism behind the effect of RANK-c isoform on NF-kB activation through RANK, we transiently transfected 293T cells with the combination of wt HA-RANK with GFP-tagged RANK-c, or each molecule alone. Forty-eight hours after transfection, cells were stained with an anti-HA antibody and visualized under an IF microscope. Interestingly, in permeabilized cells that were double positive for HA-RANK and GFP-RANK-c expression, both transfectants always co-localized in the cytoplasm and HA-RANK did not show any membrane staining (Figure 5A). However, in cells that were only HA-RANK-positive there was a clear membrane staining pattern and in cells that were only GFP-RANK-c-positive there was only cytoplasmic localization of the tagged protein. Furthermore, when the same staining procedure was performed on non-permeabilized transfected cells, membrane HA-RANK never co-localized with GFP-RANK-c-positive cells indicating that RANK-c inhibits the translocation of the wild type receptor to the cell membrane (Figure 5B and Figure S5A in Additional file 6). In addition, to further support a possible RANK-c-induced inhibition of wt RANK membrane translocation, we performed flow cytometry analysis on 293T transfected cells with wt RANK or combinations of wt RANK, RANK-b and RANK-c. Data analysis of wt RANK and RANK-c combinations, confirms the IF results and provides additional evidence on a possible mechanism of wt RANK signaling regulation through RANK-c (Figure 5C). Furthermore, analysis of HA-wtRANK/RANK-b (Figure S5B in Additional file 6) and HA-RANK-b/RANK-c (Figure S5C in Additional file 6) indicates that the inhibition of wild type RANK translocation to the cell membrane is RANK-c-specific.

These results, in conjunction with the notion that wt RANK self-associates on the cell membrane either when overexpressed or upon RANKL stimulation [32] may indicate one possible mechanism by which RANK-c isoform can regulate RANK signaling through reducing the levels of signal-transducible, self-associated receptor on the cell surface.

### Overexpression of RANK-c inhibits the RANKL and wild type RANK pro-survival effect after doxorubicin treatment

To characterize the cellular effects of RANK-c, either alone or in conjunction with the wild type receptor or RANKL treatment, we assessed cell viability after doxorubicin addition in transiently transfected MDA-MB-231 and 293T cells.

**Figure 6:**
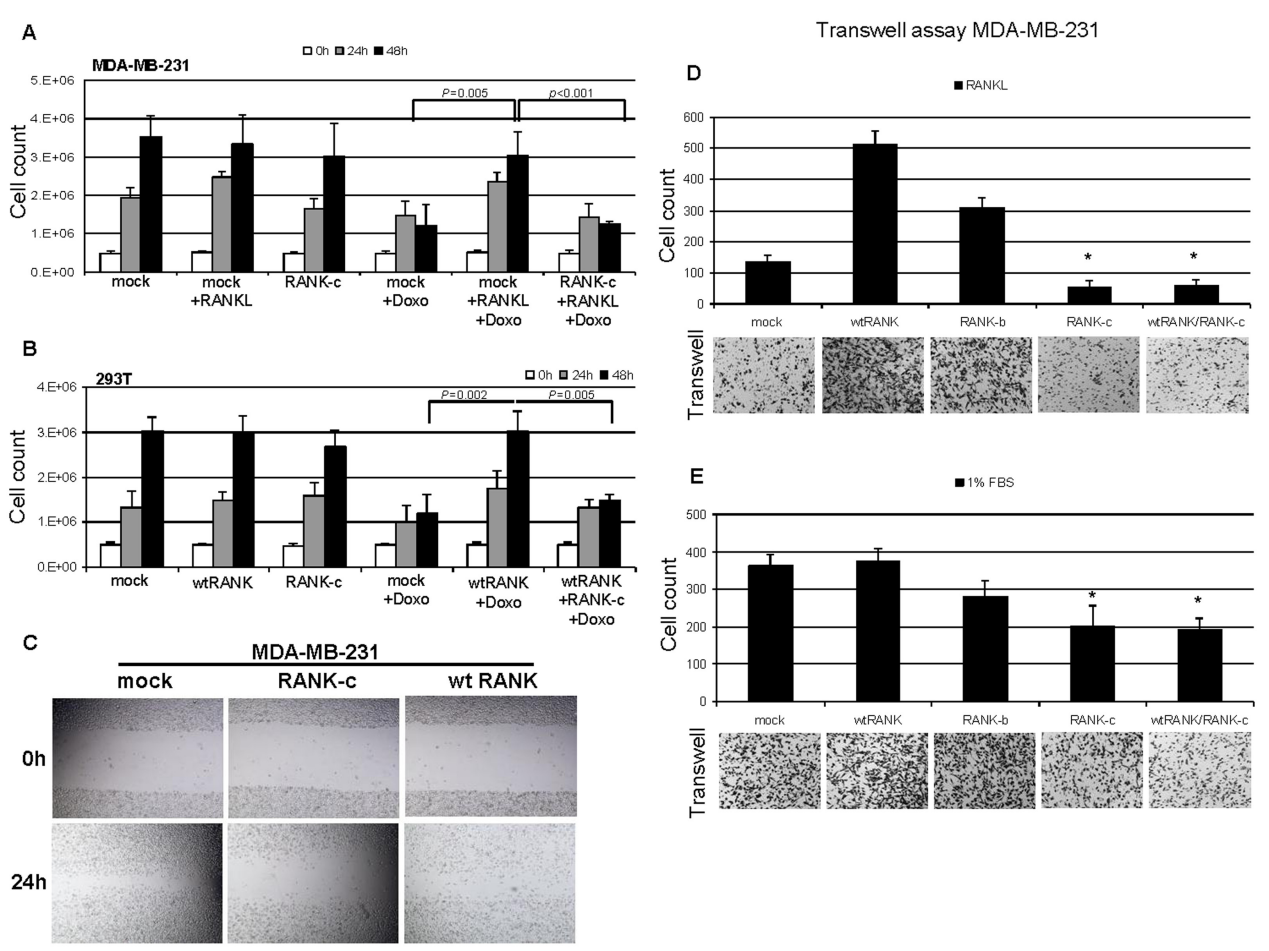
**Receptor activator of NF-kB (RANK)-c regulates wild type (wt) RANK-dependent survival and inhibits cell motility and migration**. **A **and **B**. MTT proliferation/viability assays for MDA-MB-231 and 293T transfected cells, after the addition of doxorubicin (Doxo) and/or RANK ligand (RANKL). Plasmid transfections and doxorubicin or RANKL treatments as indicated. **C**. RANK-c reduces the motility of MDA-MB-231 cells, compared to both wt RANK and mock-transfected cells as assessed by the wound-healing assay. **D **and **E**. Migration of mock- and isoform-transfected MDA-MB-231 cells towards RANKL (1 μg/ml) or 1% FBS was assessed with the use of 8-μm pore Transwell chambers. *P < 0.001 in comparison to wt RANK.

MDA-MB-231 is reported to be a RANKL-responsive breast cancer cell line [11] and as such we examined cell viability after RANKL treatment. RANKL seemed to have no effect on MDA-MB-231 cell viability and proliferation after 48 h, when compared to untreated cells under standard culturing conditions. Then, the experiment was repeated with the addition of 0.6 mg/ml doxorubicin and cell viability was assessed. Interestingly, cells treated with RANKL in the presence of doxorubicin could survive and proliferate in contrast with untreated MDA-MB-231 cells, which in the presence of doxorubicin failed to survive and proliferate. Next, we tested how RANK-c overexpression in MDA-MB-231 cells could affect the RANKL pro-survival effect observed, after doxorubicin addition. RANK-c seems to be able to significantly (*P *< 0.001) attenuate the pro-survival effect produced by RANKL treatment in the MDA-MB-231 breast cancer cell line (Figure 6A).

Furthermore, cell viability after addition of doxorubicin (0.2mg/ml) was assessed in transiently transfected 293T cells. The wt RANK- transfected cells were able to endure doxorubicin cytotoxicity and this phenomenon was reversed when wt RANK and RANK-c were co-expressed (Figure 6B). In addition, cell death (necrosis, late apoptosis and early apoptosis) after doxorubicin addition in 293T cells was assessed through annexin-V/PI staining. Total cell death was observed in approximately 22% of mock-transfected 293T cells, whilst the introduction of wt RANK receptor cDNA significantly (*P *< 0.001) reduced doxorubicin-induced toxicity 3-fold. The effect of the wild type receptor on cell viability could be partially reversed when the two forms of RANK were co-expressed (*P *= 0.005) (Figure S6A in Additional file 7).

These findings seem to be in line with results shown in Figure 4, where NF-kB activation through RANK receptor could be inhibited in the presence of RANK-c isoform, thus down-regulating the pro-survival effect of NF-kB activation.

### Overexpression of RANK-c inhibits motility and cell migration

The motility of mock-transfected, wt RANK and RANK-c transfected MDA-MB-231 and 293T cells, was assessed with the wound healing assay. Mock-transfected and wt-transfected cells were capable of closing the wound almost completely within 24 h, whereas RANK-c expressing cells were less motile and showed significantly slower rates of wound-healing (Figure 6C and Figure S6B in Additional file 7). Furthermore, the treatment with RANKL did not alter cell motility compared to mock and wt-transfected cells while the combination of wt RANK/RANK-c transfection produced similar results to RANK-c transfection on MDA-MB-231 cell motility (Figure S6C in Additional file 7).

To evaluate the role of RANK isoforms in cell migration, single-cell motility was assayed using uncoated transwells and the highly metastatic breast cancer cell line MDA-MB-231. Plasmid constructs directing the expression of mock, wt RANK, RANK-b (the membrane-bound form of RANK-c) and RANK-c were transiently transfected into MDA-MB-231 cells. Thirty-six hours post-transfection, cells were loaded in the upper chamber of the transwell and RANKL or 1% FBS was used in the lower chamber as chemoattractant. Overexpression of wt RANK and RANK-b increased MDA-MB-231 migration compared with mock-transfected cells, when recombinant RANKL was used as chemoattractant in the lower chamber. Furthermore, RANK-c-transfected cells showed significantly (*P *< 0.001) lower migration rates, compared to both mock-transfected and isoform (wt RANK and RANK-b)-transfected cells (Figure 6D). Interestingly, partial inhibition of migration was also observed for RANK-c transfected cells, even towards 1% FBS medium, indicating a possible role for this isoform in cytoskeleton organization and cell motility (Figure 6E). Finally, co-expression of wt RANK with RANK-c in MDA-MB-231 cells reduced migration rates, both towards RANKL and 1% FBS (Figure 6D and 6E), indicating that RANK-c expression could regulate the wild type receptor effect.

## Discussion

The RANK/RANKL system is emerging as a key player in the normal physiology of the mammary gland [5] with significant implications in breast cancer initiation [8,33,34], progression [9,34] and metastasis [10,11,34]. Furthermore, the RANK/RANKL pathway seems to regulate, in conjunction with sex hormones, proliferation and renewal of MaSC (mammary stem cell) pool under physiological conditions in normal mammary tissue but also in breast cancer [3,4].

While this is the first report on identification of the RANK receptor isoforms, there are already three identified RANK ligand isoforms with differential expression patterns in bone and thymus [35]. Furthermore, RANK ligand has been the target of extensive research during the last decade, both at preclinical and clinical level [36,37]. In contrast, little is known about RANK receptor function and regulation at the molecular and cellular level, despite its wide tissue expression profile and its capacity to regulate divergent organs/functions (for example, lymph node development, bone remodeling and thermoregulation) [12,16,38].

In this study we aimed to elucidate RANK regulation at the post-transcriptional level through alternative splicing, and further investigate the functional implications of the existence of such variants on the RANK/RANKL pathway. We were able to identify three full-length *TNFRSF11A *gene variants differentially expressed between tissues and cell lines. Interestingly, variant *TNFRSF11A_Δ7,8,9 *was highly upregulated in human breast cancer samples showing an inverse correlation with disease severity. The upregulation of the *TNFRSF11A_Δ7,8,9 *variant observed in breast cancer tissues may reflect either major changes in the mammary cell compartment at the molecular level [4,39] and/or changes in the tumor microenvironment, such as immune cell infiltration (T cells, macrophages, and so on) [39], occurring from early stages of breast tumorigenesis. There is also the intriguing possibility that the novel RANK variants, identified in this study, and especially *TNFRSF11A_Δ7,8,9 *have roles in the regulation of mammary stem cell and tumor-initiating cell (mammary cancer stem cells) expansion and renewal capacity, through the NF-kB machinery [40,41].

It is well established that many of the biological effects exerted by RANK are mediated through NF-kB signaling [42]. Because RANK variants are present in combination with the wild type receptor in most cell lines used in this study, we speculate a possible interaction between isoforms in regulating RANK signaling. Indeed, expression of isoform combinations in 293T cells identified RANK-c (*TNFRSF11A_Δ7,8,9*) as a putative dominant negative regulator of wt RANK-induced NF-kB activation. Furthermore, our data indicate that this effect is specific for RANK-c, and isoform RANK-b (*TNFRSF11A_Δ8,9)*, which contains exon 7 (RANK transmembrane domain) and represents the membrane-bound form of RANK-c, is incapable of inhibiting NF-kB activation by RANK. In addition, RANK-b was found to be able to activate NF-kB in contrast to RANK-a, which seems to act as an inactive receptor, though incapable of inhibiting RANK signaling. The capacity of RANK-b to activate NF-kB could be attributed to the retention of 93 amino acid residue of cytoplasmic tail (523-616aa of the wt RANK), encompassing important signaling motifs such as -IVVY- (activation of unknown signaling pathway) [43] and -PVQEET-, -PVQEQG- (activation of NF-kB and p38) [44]. Nevertheless, and despite the extensive work done on the intracellular part of RANK through a panel of truncation constructs [43-45], the exact intracellular molecules that are able to interact with the novel RANK isoforms (for example TRAFs) and mediate their functions, are still to be identified.

The distinctive difference between RANK-b and RANK-c is the exclusion of exon 7 from the latter, affecting the localization of the protein (cytoplasmic versus membrane bound). Hence we sought to study the localization of the wild type receptor in conjunction with isoform RANK-c. Indeed, when both proteins were expressed in the same cell, the presence of RANK-c isoform seemed to affect the capacity of the wild type receptor to translocate to the cell surface. A similar effect has been previously reported for CD40 variants and wt CD40 receptor [28].

The RANK receptor, through its interaction with RANKL, regulates cell proliferation, survival and differentiation in many cell types [5,15,46]. In addition, lately, the RANK/RANKL system has been identified as having pro-tumorigenic and pro-metastatic activities in various human malignancies and specifically in breast cancer [36]. Our experimental data identified the novel isoform RANK-c as a regulator of RANK/RANKL-dependent survival through a direct effect on wt RANK-dependent NF-kB activation and also as an inhibitor of cell migration through an indirect mechanism that is as yet unidentified.

The observed reduction of cell viability, when co-transfecting wt RANK with RANK-c, can be attributed to the downregulation of NF-kB. However, the inhibitory effect on cell migration observed for RANK-c, independently of both wt RANK transfection and RANKL stimuli, cannot be exclusively ascribed to NF-kB regulation. A possible explanation is provided by Armstrong and co-workers [47] who have reported on a RANK deletion construct (RANK Δ340-421) that lacks part of exon 9, resembling both RANK-b and RANK-c identified in the present study, which upon transfection was able to disrupt c-Src and c-Cbl localization, altering cytoskeleton organization in osteoclasts. A similar mechanism could be responsible for the inhibition of migration observed for 293T cells and MDA-MB-231 breast cancer cells in wound-healing and transwell assays in this study. In addition, the lower expression levels observed for variant *TNFRSF11A_Δ7,8,9 *(RANK-c) in high-grade, as opposed to low-grade breast tumors in conjunction with the inhibitory effects on cell migration, gives rise to the possibility that RANK-c could act as a novel suppressor of metastasis. Nevertheless, further work is needed to fully elucidate this newly characterized capacity of RANK-c isoform.

An important finding of this study is the upregulation of *TNFRSF11A_Δ7,8,9 *(RANK-c) in grade 1 and 2 breast cancer tissue samples in contrast to grade 3 tissue. This finding, independent of the cellular function of RANK-c isoform, in conjunction with the structure of RANK-c lacking a transmembrane domain and the identification of this isoform in supernatants of transfected 293T cells, indicates the possibility of a novel biomarker for breast cancer that is related to disease severity and/or metastasis but most importantly could be secreted.

**Figure 7:**
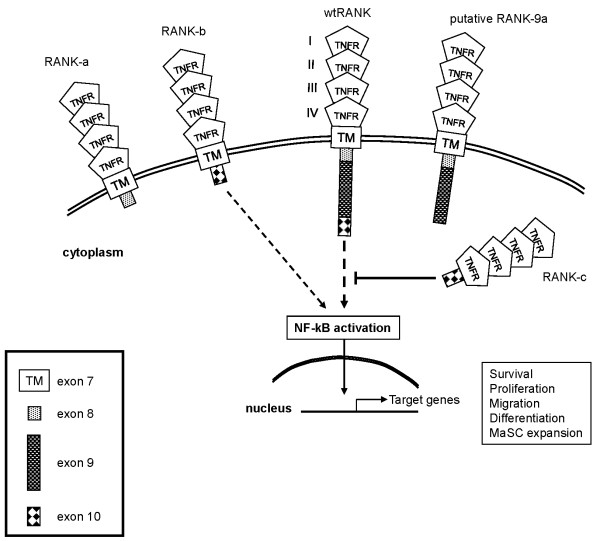
**Proposed model of the possible function of receptor activator of NF-kB (RANK) isoforms in fine-tuning downstream signaling**. The signaling interactions of RANK isoforms can produce divergent results on survival, differentiation, proliferation and migration depending on expression pattern and cell type.

Finally, the identification, for the first time, of multiple *TNFRSF11A *transcripts provides evidence for a more complex regulation for the RANK/RANKL system at the receptor level [48] and a sensitive mechanism for the receptor to fine-tune downstream signaling upon RANKL ligation, differentially affecting cell fate (Figure 7).

## Conclusions

The RANK receptor is a TNF superfamily member with critical roles in mammary, bone and lymph node development, but also in breast cancer initiation progression and metastasis. In this study, we identify for the first time, alternatively spliced variants of the RANK receptor and provide evidence of a complex regulatory pathway for RANK receptor signaling through its variants. The existence of multiple variants implies a more complex function of RANK in those cells (for example, MaSCs, osteoclasts), tissues (for example, mammary, bone) and tumors (for example, breast tumors) whose survival, development and differentiation depends on expression of the RANK receptor. Moreover, we were able to show that isoform RANK-c is upregulated in breast cancer samples and seems to correlate inversely with histological grade. Furthermore, we provide experimental data that RANK-c is a dominant negative regulator of NF-kB activation through wt RANK and at the same time it is a potent inhibitor of RANK-induced cell survival and cell migration. Finally, the possibility of RANK-c being a secreted molecule in conjunction with the fact that RANK-c is upregulated in breast cancer, could lead to the development of a novel biomarker for breast cancer initiation, progression and metastasis.

## Abbreviations

FBS: Fetal bovine serum; FHS: Fetal horse serum; GFP: Green Fluorescent Protein; MaSC: Mammary stem cell; M-CSF: Macrophage colony-stimulating factor; PBMCs; Peripheral blood mononuclear cells; RANK: Receptor activator of NF-kB; RANKL: Receptor activator of NF-kB ligand; TNFRSF11A: Tumor necrosis factor receptor superfamily, member 11a; TRAF: TNF receptor associated factor.

## Competing interests

The authors declare that they have no competing interests.

## Authors' contributions

ADP and CS designed and executed all the experimental procedures and wrote the manuscript. HPK supervised the study, edited and revised the manuscript. All authors read and approved the final manuscript.

## Supplementary Material

Additional file 1 **A table showing primer sets and PCR cycling conditions employed in the present study**. Click here for file

Additional file 2 **A figure showing the identification of the novel exon 9a of the tumor necrosis factor receptor superfamily, member 11a (*TNFRSF11A*) gene**. **A**. Intron-exon boundaries of the novel exon 9a (EMBL: HE659518). The novel exon has 148 bp length and is placed 9,772 bp downstream of exon 9 and 5346 bp upstream of exon 10 (starts at nucleotide 58943 of NG_008098.1). **B**. Exon 9a spliced in between exons 9 and 10. A stop codon (TAA) is encoded in the five first nucleotides of exon 9a when translated *in silico*, giving rise to a 523aa truncated form of RANK. **C**. Schematic representation of RANK protein and the putative truncated form (RANK-9a) lacking residues corresponding in exon 10. **D**. Graphical representation of the putative *TNFRSF11A*_exon9a gene. Black circle indicates the position of the stop codon. Click here for file

Additional file 3 **A figure showing Western blot analysis and immunofluorescense staining for receptor activator of NF-kB (RANK) receptor isoforms in transfected and non-transfected cells**. A. Western blot of transfected 293T cells with the indicated plasmid constructs. Antibody AF683 (R&D) was used for recognition of the extracellular part of RANK isoforms. **B**. Western blot of transfected 293T cells with the indicated plasmid constructs, using antibody sc-9072 recognizing intracellular amino acids 317-616. The sc-9072 Ab is unable to detect RANK-a (lacking intracellular aa 261-616), but identifies RANK-c which retains aa 523-616. **C**. Western blot of supernatants from 293T cells transfected with RANK-c or GFP-RANK-c as indicated. RANK-c appears as a double band (AF683, R&D). **D**. Western blot of RANK-c transfected 293T cells and cell lysates from MCF7 and MDA-MB-231 depicting (i) the RANK-c protein migrating as a double band at approximately 40 to 45 kDa (AF683, R&D). **E**. Western blot from the cytoplasmic fraction of the indicated cell lines depicting putative RANK-c protein migrating at 40 to 45 kDa and possible other RANK variants (AF683, R&D). **F**. Total Protein Western Blot (W1235086, Biochain)from human breast cancer tissues, collected from three different donors (invasive ductal carcinoma grade 2, invasive lobular carcinoma grade 2) (AF683, R&D). **G**. Immunofluorescent-stained 293T cells transfected with RANK plasmid isoforms (upper panel) and green fluorescent protein (GFP)-RANK-c (lower panel). Antibody AF683 was used for recognition of the extracellular part of RANK isoform, while sc-9072 was used for recognition of the intracellular part of RANK (aa 317-616). Click here for file

Additional file 4 **Figure showing mRNA expression of tumor necrosis factor receptor superfamily, member 11a (*TNFRSF11A*) variants in a panel of cell lines and *TNFRSF11A_Δ7,8,9 *in formaldehyde-fixed paraffin-embedded (FFPE) samples immunohistochemically diagnosed as ductal invasive breast carcinoma**. **A**. RT-PCR amplification of *TNFRSF11A *variants using primers P1 and P2. *TNFRSF11A *variants follow a different pattern of expression in a panel of cell lines. **B**. Correlation of *TNFRSF11A_Δ7,8,9 *mRNA levels with histological grade and proliferation index. **C**. Immunoexpression of protein markers and histological grade of 21 FFPE breast carcinoma samples analyzed in the present study. Click here for file

Additional file 5 **A figure showing results of the luciferase assay for receptor activator of NF-kB (RANK) isoform-induced NF-kB activation**. **A**. Luciferase assay depicting the downregulation of NF-kB activation produced by the combined transfection of wild type (wt) RANK and RANK-c, but not with any other combination. *P < 0.001 between wt RANK- and RANK-c-treated (RANKL) and untreated. **B**. Co-transfection of 293T cells with wt RANK and RANK-b (as indicated), does not affect NF-kB activation. **C**. Co-transfection of 293T cells with RANK-b and increasing amounts of RANK-c. RANK-c seems able to downregulate NF-kB activation, though not to the same extent as for wt RANK. RT-PCR on total RNA isolated from transfected cells, serving as a transfection control (right panel). **D**. Co-transfection of 293T cells with wt RANK and increasing amounts of green fluorescent protein (GFP)-RANK-c. The GFP tagged RANK-c seems to have the same effect on wt receptor NF-kB activation, as does RANK-c. RT-PCR on total RNA isolated from transfected cells, serving as a transfection control (right panel). Click here for file

Additional file 6 **A figure showing flow cytometry analysis of 293T cells, transfected with combinations of various receptor activator of NF-kB (RANK) isoforms**. **A**. Non-permeabilized transfected 293T cells with both human influenza hemagglutin epitope (HA)-RANK and green fluorescent protein (GFP)-RANK-c plasmids. HA molecules were visualized with the use of anti-HA from Santa Cruz (sc-57592). **B**. Flow cytometry analysis of 293T cells co-transfected with HA-wild type (wt) RANK and RANK-b, indicating that RANK-b is unable to inhibit HA-wt RANK translocation to the cell membrane. **C**. 293T cells were transfected with HA-RANK-b alone or in combination with RANK-c. Flow cytometry analysis demonstrates that RANK-c is incapable to inhibit HA-RANK-b translocation to the cell surface. Click here for file

Additional file 7 **A figure showing the flow cytometry viability assay and wound-healing assay for 293T and MDA-MB-231 cells after transfection with the indicated receptor activator of NF-kB (RANK) isoform**. **A**. 293T cells were plated (1 × 10^5^) in twelve-well plates and transiently transfected with the respective constructs. At 24 h post-transfection doxorubicin HCL (Ebewe Pharma, Austria) (0.2 mg/ml) was added for another 24 h. At the end of the 24 h-doxorubicin-incubation, cells were washed twice with PBS and trypsinized. Cells were stained for annexin V-FITC and propidium iodide (PI) (rh Annexin V/FITC kit, Bender MedSystems) and were immediately analyzed by flow cytometry (EPICS-XL, Coulter) according to manufacturer's instructions. Double-positive cells were considered as late apoptotic, PI single-positive cells or Annexin V single-positive cells were considered necrotic and early apoptotic, respectively. All treatments were done in duplicate and experiments were repeated at least twice. **B**. RANK-c reduces the motility of 293T cells, compared to both wild type (wt) RANK and mock-transfected cells as assessed by the wound-healing assay. **C**. Wound healing assay of MDA-MB-231 cells either transfected with wt RANK/RANK-c plasmids or treated with RANKL. Click here for file
