# Correction to: Identification of novel human RANK isoforms generated through alternative splicing. Implications in breast cancer cell survival and migration

**DOI:** 10.1186/s13058-018-0985-z

**Published:** 2018-06-07

**Authors:** Anastasios D. Papanastasiou, Chaido Sirinian, Haralabos P. Kalofonos

**Affiliations:** Clinical and Molecular Oncology Laboratory, Division of Oncology, Department of Medicine, University of Patras, 26504 Rion, Greece

## Correction

After the publication of this article [1] we noticed that in Fig. 1, the gel images (1A and 1B lower panel) were incorrect. The corrected Fig. 1 is presented below. The correction does not affect in any way our results and conclusions.

**Fig. 1:**
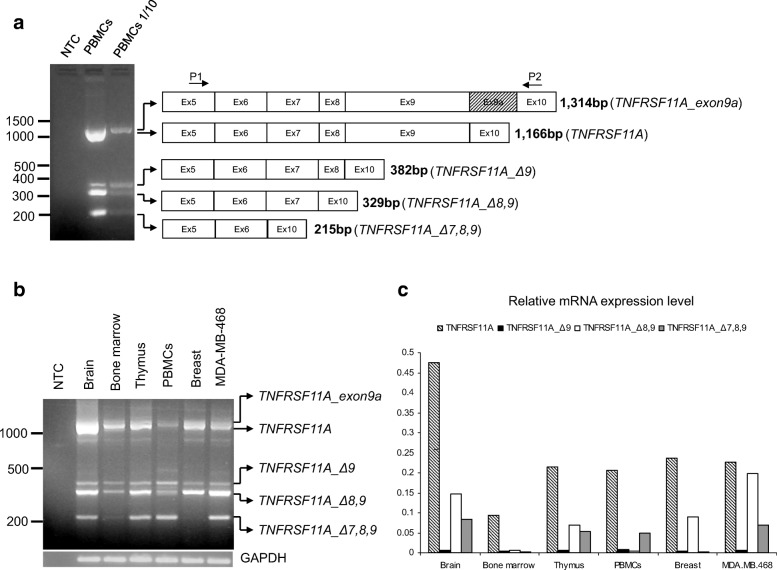
**a**. Agarose gel electrophoresis of the PCR products using primers P1 and P2 on cDNA from human PBMCs and the graphical representation of the splice products identified. **b**. Agarose gel electrophoresis of PCR products depicting *TNFRSF11A* variant distribution from a panel of human normal tissue RNAs and MDA-MB-468 as a control. **c**. Quantitative RT-PCR of the novel splice variants and wild type RANK from a panel of human normal tissue RNAs. Data normalization was carried out against *GAPDH* housekeeping gene.
