# Supplementary material for: Identification of novel human receptor activator of nuclear factor-kB isoforms generated through alternative splicing: implications in breast cancer cell survival and migration
Source: Breast Cancer Res. 2012 Jul 23;14(4):R112. doi: 10.1186/bcr3234 (PMC3680950; doi:10.1186/bcr3234)
Supplement: Additional file 1 — A table showing primer sets and PCR cycling conditions employed in the present study. [file bcr3234-S1.DOC]

Supplementary Table 1. Primers used in the present study.

| P1 | TTCTCTGATGCCTTTTCCTCC | Exon 5 |
| --- | --- | --- |
| P2 ^a,b^ | ATGATGTCGCCCTTGAAGTTC | Exon 10 |
| P3 | CTGACTGAAGCCCATAGCAC | Forw. Exon 9a |
| P4 | CGCCCGCCAGCCTGTCCCG | Exon 1 |
| P5 | AGAGGCTGCGGTGCTGCCC | Exon 10 |
| P6 | TTAAGCTTATGGCCCCGCGCGCCC | HindIII |
| P7 | AAGGATCCAATCAAGCCTTGGCCCCGCC | BamHI |
| P6RT ^c^ | CATCATGGGACAGAGAAATCC | Forw. TNFRSF11A_Δ7,8,9 |
| P7RT ^c^ | TTCCAGTCACATTTCCATTTGG | Rev. TNFRSF11A_Δ7,8,9 |
| P8RT ^b^ | GGAAAGCACTCACAGGAAATG | Forw. TNFRSF11A_Δ8,9 |
| P9RT ^a^ | TAAGTGGAGATAAGGAAATGTG | Forw. TNFRSF11A_Δ9 |
| P10RT^d^ | CTTCTCTTCGCGTCTGTGG | Exon 7 |
| P11RT^d^ | ATGCTCCCTGCTGACCAAAG | Exon 9 |

Set of primers used to applify the TNFRSF11A_Δ9^a^, TNFRSF11A_Δ8,9^b^ and TNFRSF11A_Δ7,8,9 ^c^ variants by qRT-PCR (94 ^o^C for 30sec, 58 ^o^C for 30sec, 72 ^o^C for 1sec). Set of primers to amplify TNFRSF11A^d^ by qRT-PCR (94 ^o^C for 30sec, 60 ^o^C for 30sec, 72 ^o^C for 1sec).
