# Supplementary material for: Identification of novel human receptor activator of nuclear factor-kB isoforms generated through alternative splicing: implications in breast cancer cell survival and migration
Source: Breast Cancer Res. 2012 Jul 23;14(4):R112. doi: 10.1186/bcr3234 (PMC3680950; doi:10.1186/bcr3234)
Supplement: Additional file 2 — A figure showing the identification of the novel exon 9a of the tumor necrosis factor receptor superfamily, member 11a (TNFRSF11A) gene. A. Intron-exon boundaries of the novel exon 9a (EMBL: HE659518). The novel exon has 148 bp length and is placed 9,772 bp downstream of exon 9 and 5346 bp upstream of exon 10 (starts at nucleotide 58943 of NG_008098.1). B. Exon 9a spliced in between exons 9 and 10. A stop codon (TAA) is encoded in the five first nucleotides of exon 9a when translated in silico, giving rise to a 523aa truncated form of RANK. C. Schematic representation of RANK protein and the putative truncated form (RANK-9a) lacking residues corresponding in exon 10. D. Graphical representation of the putative TNFRSF11A_exon9a gene. Black circle indicates the position of the stop codon. [file bcr3234-S2.PPT]

## Slide 1
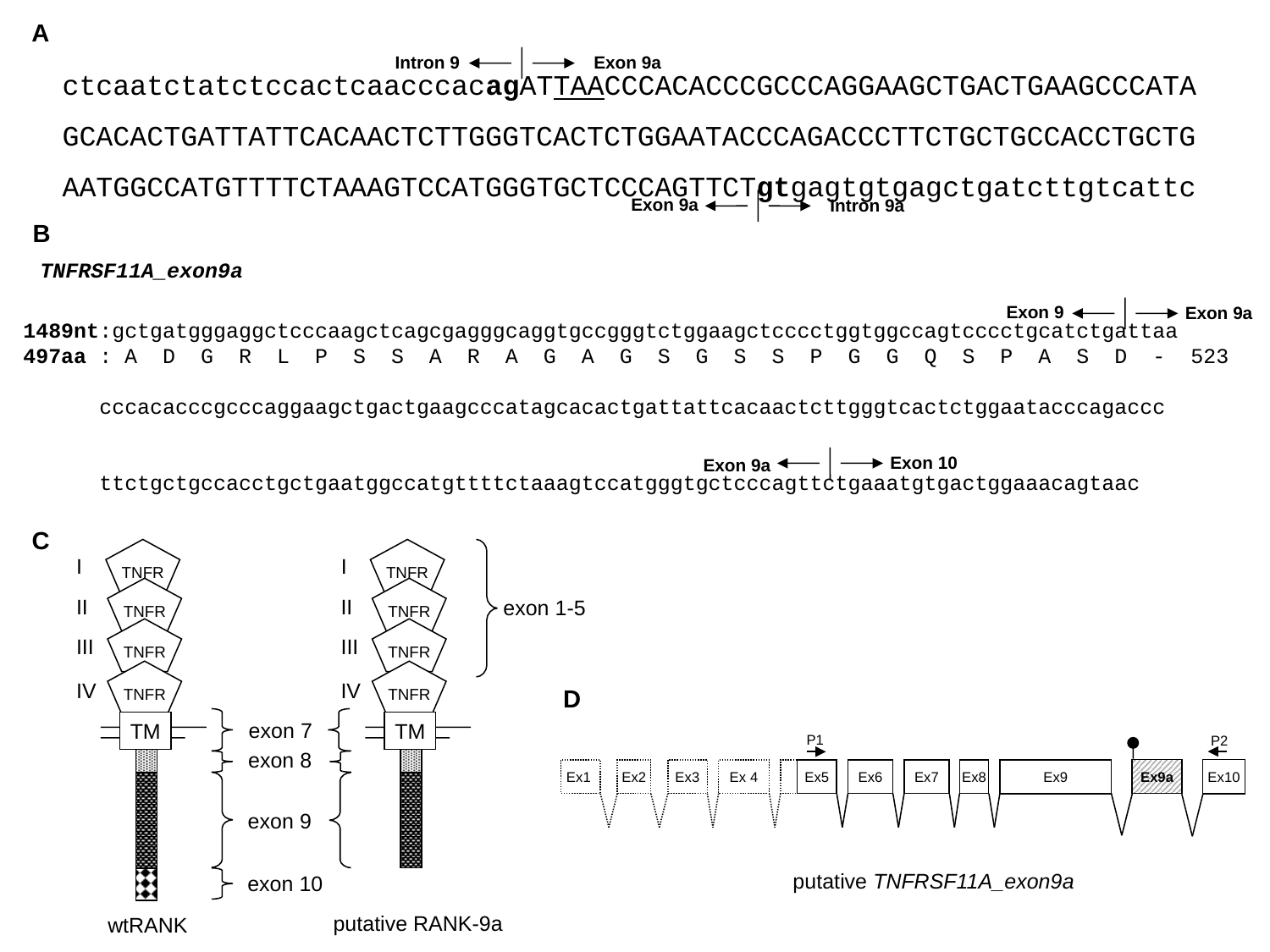

A
ctcaatctatctccactcaacccacagATTAACCCACACCCGCCCAGGAAGCTGACTGAAGCCCATAGCACACTGATTATTCACAACTCTTGGGTCACTCTGGAATACCCAGACCCTTCTGCTGCCACCTGCTGAATGGCCATGTTTTCTAAAGTCCATGGGTGCTCCCAGTTCTgtgagtgtgagctgatcttgtcattc
Intron 9
Exon 9a
Exon 9a
Intron 9a
B
 TNFRSF11A_exon9a
1489nt:gctgatgggaggctcccaagctcagcgagggcaggtgccgggtctggaagctcccctggtggccagtcccctgcatctgattaa
497aa : A D G R L P S S A R A G A G S G S S P G G Q S P A S D - 523
 cccacacccgcccaggaagctgactgaagcccatagcacactgattattcacaactcttgggtcactctggaatacccagaccc
 ttctgctgccacctgctgaatggccatgttttctaaagtccatgggtgctcccagttctgaaatgtgactggaaacagtaac
Exon 9
Exon 9a
Exon 10
Exon 9a
C
TNFR
TNFR
I
I
TNFR
TNFR
II
II
exon 1-5
TNFR
TNFR
III
III
TNFR
TNFR
IV
IV
D
exon 7
TM
TM
P1
Ex1
Ex2
Ex3
Ex 4
Ex5
Ex6
Ex7
Ex8
P2
Ex9a
Ex10
Ex9
putative TNFRSF11A_exon9a
exon 8
exon 9
exon 10
putative RANK-9a
wtRANK
