# Supplementary figures and images for: Identification of novel human receptor activator of nuclear factor-kB isoforms generated through alternative splicing: implications in breast cancer cell survival and migration
Source: Breast Cancer Res. 2012 Jul 23;14(4):R112. doi: 10.1186/bcr3234 (PMC3680950; doi:10.1186/bcr3234)

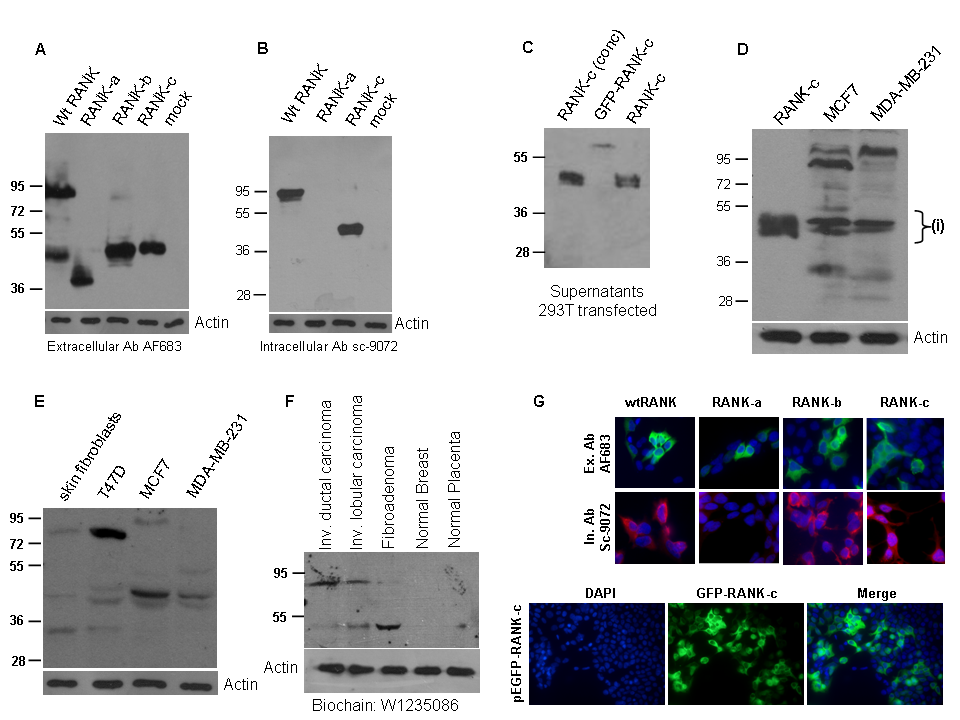

Supplement: Additional file 3 — A figure showing Western blot analysis and immunofluorescense staining for receptor activator of NF-kB (RANK) receptor isoforms in transfected and non-transfected cells. A. Western blot of transfected 293T cells with the indicated plasmid constructs. Antibody AF683 (R&D) was used for recognition of the extracellular part of RANK isoforms. B. Western blot of transfected 293T cells with the indicated plasmid constructs, using antibody sc-9072 recognizing intracellular amino acids 317-616. The sc-9072 Ab is unable to detect RANK-a (lacking intracellular aa 261-616), but identifies RANK-c which retains aa 523-616. C. Western blot of supernatants from 293T cells transfected with RANK-c or GFP-RANK-c as indicated. RANK-c appears as a double band (AF683, R&D). D. Western blot of RANK-c transfected 293T cells and cell lysates from MCF7 and MDA-MB-231 depicting (i) the RANK-c protein migrating as a double band at approximately 40 to 45 kDa (AF683, R&D). E. Western blot from the cytoplasmic fraction of the indicated cell lines depicting putative RANK-c protein migrating at 40 to 45 kDa and possible other RANK variants (AF683, R&D). F. Total Protein Western Blot (W1235086, Biochain)from human breast cancer tissues, collected from three different donors (invasive ductal carcinoma grade 2, invasive lobular carcinoma grade 2) (AF683, R&D). G. Immunofluorescent-stained 293T cells transfected with RANK plasmid isoforms (upper panel) and green fluorescent protein (GFP)-RANK-c (lower panel). Antibody AF683 was used for recognition of the extracellular part of RANK isoform, while sc-9072 was used for recognition of the intracellular part of RANK (aa 317-616). [file bcr3234-S3.BMP]

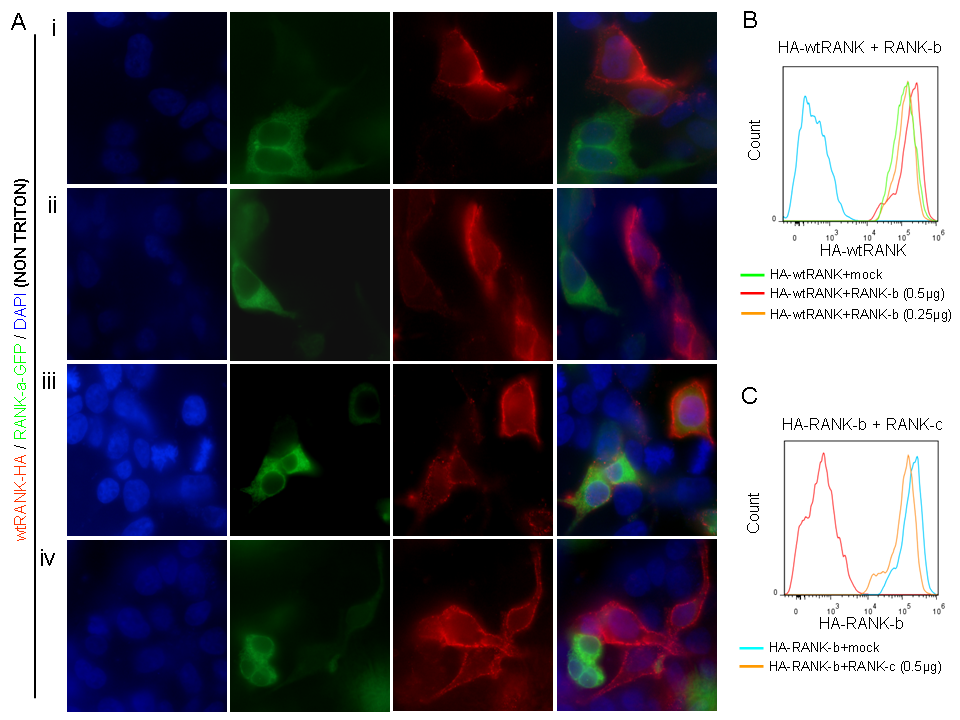

Supplement: Additional file 6 — A figure showing flow cytometry analysis of 293T cells, transfected with combinations of various receptor activator of NF-kB (RANK) isoforms. A. Non-permeabilized transfected 293T cells with both human influenza hemagglutin epitope (HA)-RANK and green fluorescent protein (GFP)-RANK-c plasmids. HA molecules were visualized with the use of anti-HA from Santa Cruz (sc-57592). B. Flow cytometry analysis of 293T cells co-transfected with HA-wild type (wt) RANK and RANK-b, indicating that RANK-b is unable to inhibit HA-wt RANK translocation to the cell membrane. C. 293T cells were transfected with HA-RANK-b alone or in combination with RANK-c. Flow cytometry analysis demonstrates that RANK-c is incapable to inhibit HA-RANK-b translocation to the cell surface. [file bcr3234-S6.BMP]
