# Supplementary material for: Identification of novel human receptor activator of nuclear factor-kB isoforms generated through alternative splicing: implications in breast cancer cell survival and migration
Source: Breast Cancer Res. 2012 Jul 23;14(4):R112. doi: 10.1186/bcr3234 (PMC3680950; doi:10.1186/bcr3234)
Supplement: Additional file 4 — Figure showing mRNA expression of tumor necrosis factor receptor superfamily, member 11a (TNFRSF11A) variants in a panel of cell lines and TNFRSF11A_Δ7,8,9 in formaldehyde-fixed paraffin-embedded (FFPE) samples immunohistochemically diagnosed as ductal invasive breast carcinoma. A. RT-PCR amplification of TNFRSF11A variants using primers P1 and P2. TNFRSF11A variants follow a different pattern of expression in a panel of cell lines. B. Correlation of TNFRSF11A_Δ7,8,9 mRNA levels with histological grade and proliferation index. C. Immunoexpression of protein markers and histological grade of 21 FFPE breast carcinoma samples analyzed in the present study. [file bcr3234-S4.PPT]

## Slide 1
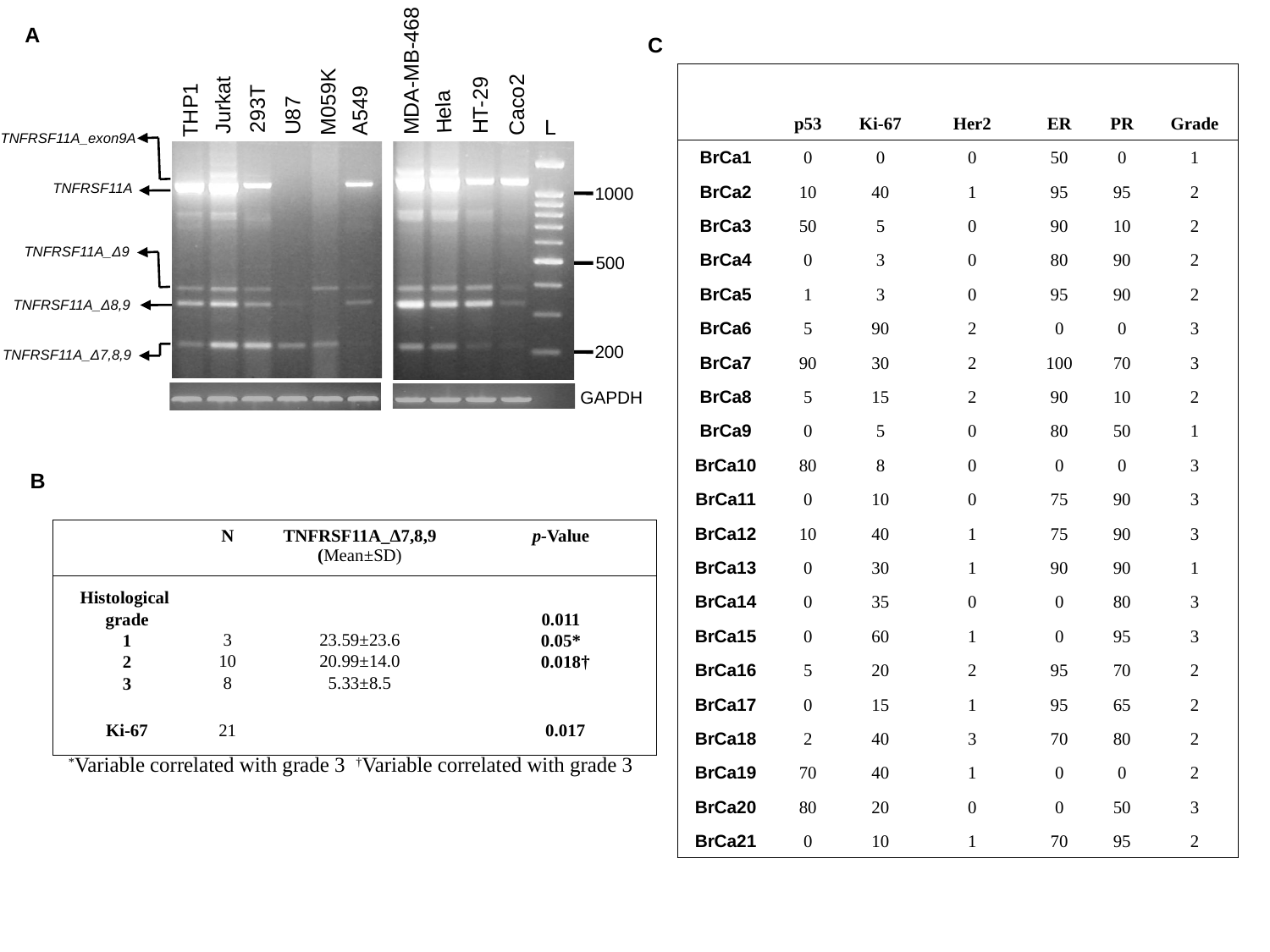

A
C
MDA-MB-468
| | p53 | Ki-67 | Ηer2 | ER | PR | Grade |
| --- | --- | --- | --- | --- | --- | --- |
| BrCa1 | 0 | 0 | 0 | 50 | 0 | 1 |
| BrCa2 | 10 | 40 | 1 | 95 | 95 | 2 |
| BrCa3 | 50 | 5 | 0 | 90 | 10 | 2 |
| BrCa4 | 0 | 3 | 0 | 80 | 90 | 2 |
| BrCa5 | 1 | 3 | 0 | 95 | 90 | 2 |
| BrCa6 | 5 | 90 | 2 | 0 | 0 | 3 |
| BrCa7 | 90 | 30 | 2 | 100 | 70 | 3 |
| BrCa8 | 5 | 15 | 2 | 90 | 10 | 2 |
| BrCa9 | 0 | 5 | 0 | 80 | 50 | 1 |
| BrCa10 | 80 | 8 | 0 | 0 | 0 | 3 |
| BrCa11 | 0 | 10 | 0 | 75 | 90 | 3 |
| BrCa12 | 10 | 40 | 1 | 75 | 90 | 3 |
| BrCa13 | 0 | 30 | 1 | 90 | 90 | 1 |
| BrCa14 | 0 | 35 | 0 | 0 | 80 | 3 |
| BrCa15 | 0 | 60 | 1 | 0 | 95 | 3 |
| BrCa16 | 5 | 20 | 2 | 95 | 70 | 2 |
| BrCa17 | 0 | 15 | 1 | 95 | 65 | 2 |
| BrCa18 | 2 | 40 | 3 | 70 | 80 | 2 |
| BrCa19 | 70 | 40 | 1 | 0 | 0 | 2 |
| BrCa20 | 80 | 20 | 0 | 0 | 50 | 3 |
| BrCa21 | 0 | 10 | 1 | 70 | 95 | 2 |
M059K
Caco2
Jurkat
HT-29
293T
THP1
A549
Hela
U87
L
TNFRSF11A_exon9A
TNFRSF11A
1000
TNFRSF11A_Δ9
500
TNFRSF11A_Δ8,9
200
TNFRSF11A_Δ7,8,9
GAPDH
B
| | N | TNFRSF11A\_Δ7,8,9 (Mean±SD) | p-Value |
| --- | --- | --- | --- |
| Histological grade 1 2 3 | 3 10 8 | 23.59±23.6 20.99±14.0 5.33±8.5 | 0.011 0.05\* 0.018† |
| Ki-67 | 21 | | 0.017 |
*Variable correlated with grade 3 †Variable correlated with grade 3
