# Supplementary material for: Identification of novel human receptor activator of nuclear factor-kB isoforms generated through alternative splicing: implications in breast cancer cell survival and migration
Source: Breast Cancer Res. 2012 Jul 23;14(4):R112. doi: 10.1186/bcr3234 (PMC3680950; doi:10.1186/bcr3234)
Supplement: Additional file 5 — A figure showing results of the luciferase assay for receptor activator of NF-kB (RANK) isoform-induced NF-kB activation. A. Luciferase assay depicting the downregulation of NF-kB activation produced by the combined transfection of wild type (wt) RANK and RANK-c, but not with any other combination. *P < 0.001 between wt RANK- and RANK-c-treated (RANKL) and untreated. B. Co-transfection of 293T cells with wt RANK and RANK-b (as indicated), does not affect NF-kB activation. C. Co-transfection of 293T cells with RANK-b and increasing amounts of RANK-c. RANK-c seems able to downregulate NF-kB activation, though not to the same extent as for wt RANK. RT-PCR on total RNA isolated from transfected cells, serving as a transfection control (right panel). D. Co-transfection of 293T cells with wt RANK and increasing amounts of green fluorescent protein (GFP)-RANK-c. The GFP tagged RANK-c seems to have the same effect on wt receptor NF-kB activation, as does RANK-c. RT-PCR on total RNA isolated from transfected cells, serving as a transfection control (right panel). [file bcr3234-S5.PPT]

## Slide 1
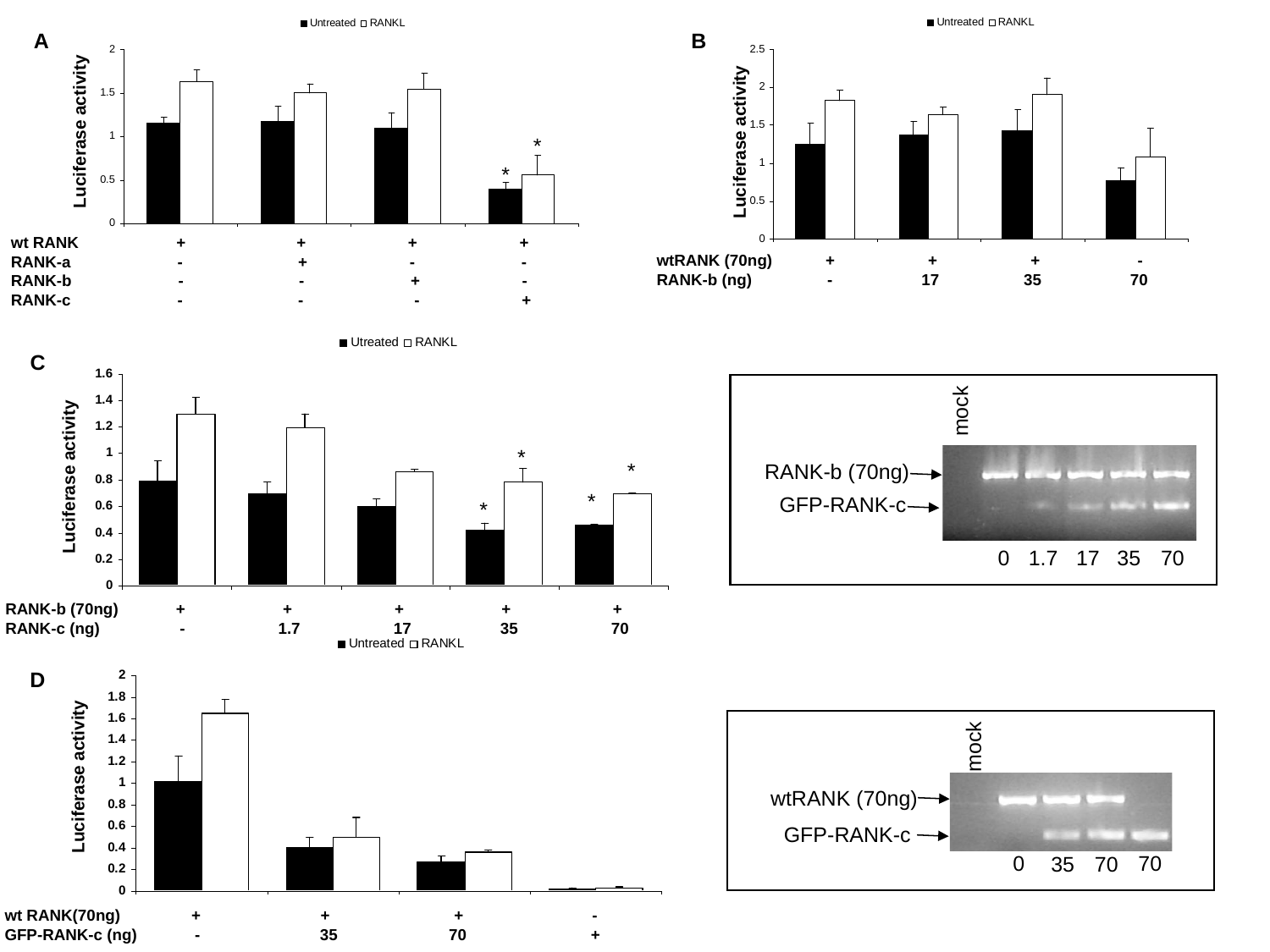

wtRANK (70ng) + + + -
RANK-b (ng) - 17 35 70
A
B
Luciferase activity
Luciferase activity
*
*
wt RANK + + + +
RANK-a - + - -
RANK-b - - + -
RANK-c - - - +
mock
RANK-b (70ng)
GFP-RANK-c
0
1.7
17
35
70
Luciferase activity
RANK-b (70ng) + + + + +
RANK-c (ng) - 1.7 17 35 70
C
*
*
*
*
D
Luciferase activity
wt RANK(70ng) + + + -
GFP-RANK-c (ng) - 35 70 +
mock
wtRANK (70ng)
GFP-RANK-c
0
70
35
70
