# Supplementary material for: Identification of novel human receptor activator of nuclear factor-kB isoforms generated through alternative splicing: implications in breast cancer cell survival and migration
Source: Breast Cancer Res. 2012 Jul 23;14(4):R112. doi: 10.1186/bcr3234 (PMC3680950; doi:10.1186/bcr3234)
Supplement: Additional file 7 — A figure showing the flow cytometry viability assay and wound-healing assay for 293T and MDA-MB-231 cells after transfection with the indicated receptor activator of NF-kB (RANK) isoform. A. 293T cells were plated (1 × 10^5^) in twelve-well plates and transiently transfected with the respective constructs. At 24 h post-transfection doxorubicin HCL (Ebewe Pharma, Austria) (0.2 mg/ml) was added for another 24 h. At the end of the 24 h-doxorubicin-incubation, cells were washed twice with PBS and trypsinized. Cells were stained for annexin V-FITC and propidium iodide (PI) (rh Annexin V/FITC kit, Bender MedSystems) and were immediately analyzed by flow cytometry (EPICS-XL, Coulter) according to manufacturer's instructions. Double-positive cells were considered as late apoptotic, PI single-positive cells or Annexin V single-positive cells were considered necrotic and early apoptotic, respectively. All treatments were done in duplicate and experiments were repeated at least twice. B. RANK-c reduces the motility of 293T cells, compared to both wild type (wt) RANK and mock-transfected cells as assessed by the wound-healing assay. C. Wound healing assay of MDA-MB-231 cells either transfected with wt RANK/RANK-c plasmids or treated with RANKL. [file bcr3234-S7.PPT]

## Slide 1
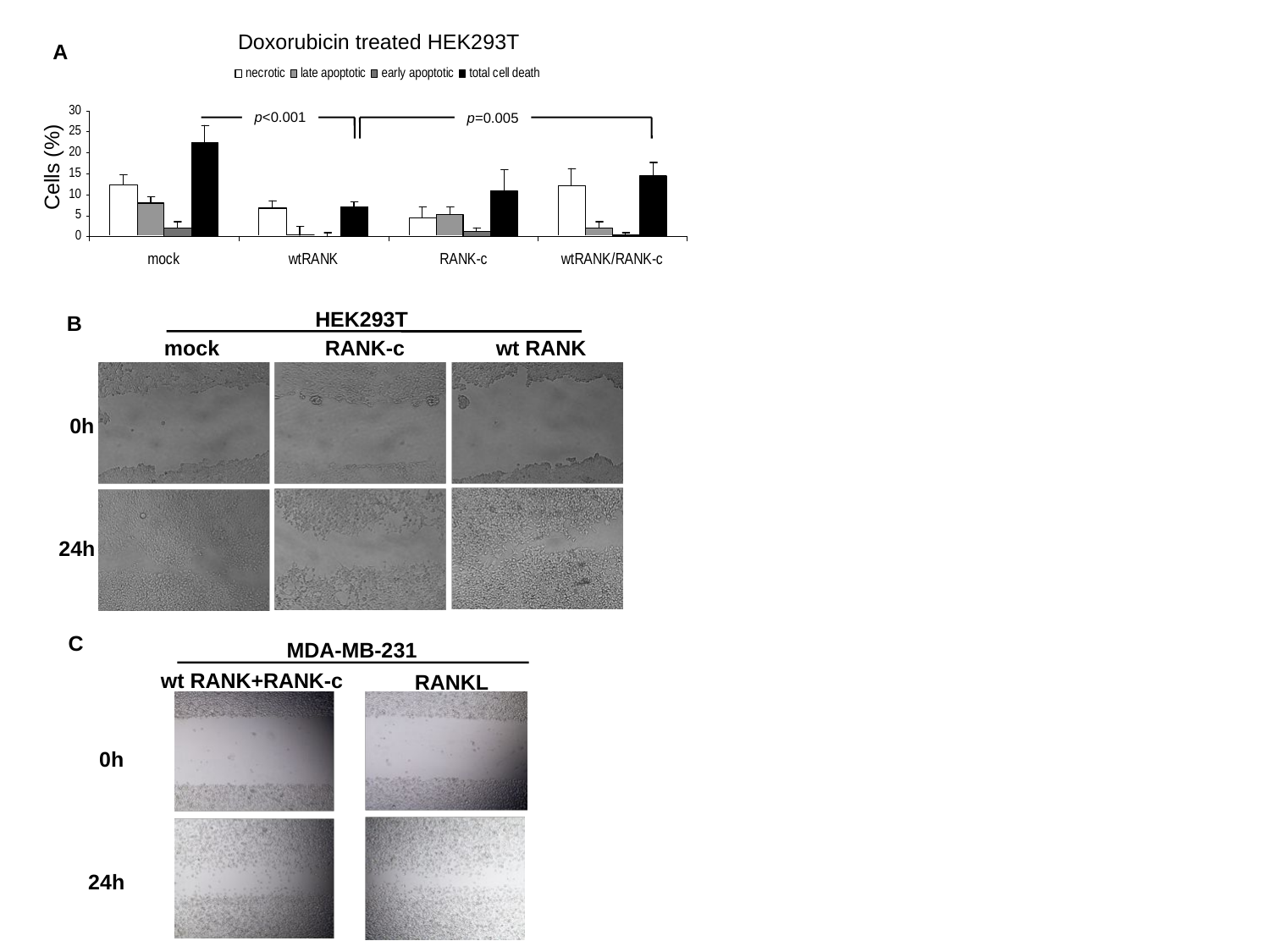

Doxorubicin treated HEK293T
A
p<0.001
p=0.005
Cells (%)
HEK293T
B
mock
RANK-c
wt RANK
0h
24h
C
MDA-MB-231
wt RANK+RANK-c
RANKL
0h
24h
